# Carbon-Coated Honeycomb Ni-Mn-Co-O Inverse Opal: A High Capacity Ternary Transition Metal Oxide Anode for Li-ion Batteries

**DOI:** 10.1038/srep42263

**Published:** 2017-02-10

**Authors:** David McNulty, Hugh Geaney, Colm O’Dwyer

**Affiliations:** 1 Department of Chemistry, University College Cork, Cork T12 YN60, Ireland; 2 Micro-Nano Systems Centre, Tyndall National Institute, Lee Maltings, Cork T12 R5CP, Ireland

## Abstract

We present the formation of a carbon-coated honeycomb ternary Ni-Mn-Co-O inverse opal as a conversion mode anode material for Li-ion battery applications. In order to obtain high capacity via conversion mode reactions, a single phase crystalline honeycombed IO structure of Ni-Mn-Co-O material was first formed. This Ni-Mn-Co-O IO converts via reversible redox reactions and Li_2_O formation to a 3D structured matrix assembly of nanoparticles of three (MnO, CoO and NiO) oxides, that facilitates efficient reactions with Li. A carbon coating maintains the structure without clogging the open-worked IO pore morphology for electrolyte penetration and mass transport of products during cycling. The highly porous IO was compared in a Li-ion half-cell to nanoparticles of the same material and showed significant improvement in specific capacity and capacity retention. Further optimization of the system was investigated by incorporating a vinylene carbonate additive into the electrolyte solution which boosted performance, offering promising high-rate performance and good capacity retention over extended cycling. The analysis confirms the possibility of creating a ternary transition metal oxide material with binder free accessible open-worked structure to allow three conversion mode oxides to efficiently cycle as an anode material for Li-ion battery applications.

Li-ion batteries continue to attract intense research aimed at improving their performance for ever more demanding energy storage applications^1^,^2^,^3^,^4^,^5^. These demands have necessitated studies into the development of active materials with higher specific capacities and enhanced cycle-lives^6^,^7^,^8^. From the perspective of anode materials for Li-ion applications, these can be broadly divided into three classes based on the reactions occurring during lithiation/delithiation namely; intercalation mode, alloying mode and conversion mode materials^9^,^10^,^11^. Conversion mode materials typically operate *via* the transformation of a transition metal oxide (e.g. Co_3_O_4_, MnO etc.) to its parent metal and Li_2_O during charging, with the metal being oxidised during discharge (for anodes)^12^,^13^. These reactions facilitate higher specific capacities than typically seen for intercalation materials and as a result, interest in the development of conversion mode materials with suitable cycle lifetimes has increased^14^,^15^.

One of the primary benefits of conversion mode materials is that they allow a great degree of tunability due to the possibility of creating mixed transition metal oxide compounds. In fact, there has been a wave of reports detailing the formation and electrochemical performance of binary transition metal oxides. The most widely investigated mixed oxide systems typically include two elements from Co, Mn, Ni, Cr, and Zn in a defined stoichiometry with a cubic/spinel type structure (e.g. MnCo_2_O_4_, NiCo_2_O_4_)^16^,^17^,^18^. For example, Li *et al*. showed impressive performance from a NiCo_2_O_4_ microsphere based anode even at high specific currents (up to 1600 mA g^−1^)^19^. Similarly, Fu *et al*. presented the fabrication of MnCo_2_O_4_ microspheres with good capacity retention up to 200 charge/discharge cycles^20^. Given these promising results, there exists scope for further investigating the mixed transition metal oxide regime with increased complexity in terms of the number of elements incorporated (i.e. moving from binary systems to ternary systems) and the morphology of the materials, aimed at further improving the performance by addressing capacity retention. Improvements in energy inefficiency from charge-discharge overpotential reduction may be feasible by engineering the structure and stabilising the cycling composition of the multi-metallic oxide phase in the cycled displacement reaction.

Three dimensional (3D) materials such as inverse opals (IOs) are very attractive architectures for the active materials of Li-ion batteries^21^. These structures offer numerous benefits such as large surface area and porosity for effective electrolyte infiltration and reduced Li-ion diffusion lengths^22^,^23^,^24^,^25^,^26^. As a result, various IOs materials have been produced as the active materials for anodes (i.e. Ge, SnO_2_)^27^,^28^ and cathodes (V_2_O_5,_ LiCoO_2_)^29^,^30^. These materials have shown improved behaviour compared to their bulk counterparts with particular improvement in their high-rate performance. We recently presented further evidence of the viability of IOs for Li-ion applications by creating a full-cell with a pre-lithiated Co_3_O_4_ IO anode and a V_2_O_5_ cathode^31^. This report confirmed the compatibility of a conversion mode anode with an intercalation mode cathode with both active materials possessing a 3D geometry.

In this report, we present the formation of a mixed Ni, Mn, Co oxide IO material for Li-ion anode applications. The material is formed from a mixed precursor solution of the respective chloride salts with a final surface stoichiometry of NiMn_1.7_Co_1.8_O_4_ (determined from X-ray photoelectron spectroscopy (XPS) analysis). This Ni-Mn-Co-O oxide material is unlike the classic lithiated NMC cathode material (LiNi_0.33_Mn_0.33_Co_0.33_O_2_)^32^,^33^,^34^ and functions as a conversion mode anode material. In order to prepare a stable, high performance anode material with high capacity, we engineered a method to form a single Ni-Mn-Co-O phase so that the nano-particulate network comprising the walls of the hierarchically porous inverse opal structure can transition from an initial ternary metal oxide (TMO) compound to an integrated matrix of three conversion mode metal oxides. Converting an initial single phase mixed TMO compound to an interconnected triple unary oxide phase system via lithiation ensures that the 3D porous networked material can be engineered as a monolithic single open structure, rather than a composite consisting of a mixture of unary metal oxides, which tend to agglomerate as individual random mixtures of particles. The electrochemical performance of the Ni-Mn-Co-O material is probed as a function of structure (by comparing nanoparticles with IOs), with or without vinylene carbonate additive in the electrolyte, and the effectiveness of carbon coating the IO structure is also investigated. This carbon-coated Ni-Mn-Co-O anode IO material is capable of stable cycling and coulombic efficiency once the final composition is reached, with high capacity retention (>400 mA g^−1^ after 100 cycles at 150 mA g^−1^ rate) for a conversion mode anode.

## Results and Discussion

**Figure 1:**
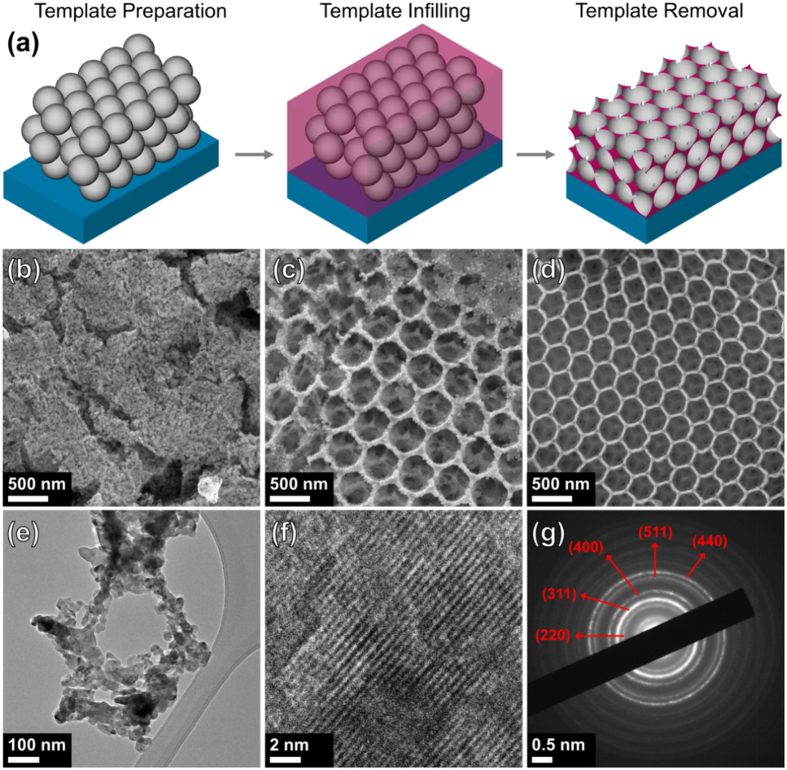
(**a**) Schematic representation of the preparation procedure for Ni-Mn-Co-O IO samples. SEM images of (**b**) Ni-Mn-Co-O nanoparticles, (**c**) standard Ni-Mn-Co-O IO and (**d**) carbon coated Ni-Mn-Co-O IO. TEM images of standard Ni-Mn-Co-O IO showing (**e**) the walls of the IO and (**f**) the lattice fringes of the Ni-Mn-Co-O NPs which comprise the walls of the IO. (**g**) Electron diffraction pattern for standard Ni-Mn-Co-O IO demonstrating its polycrystalline structure.

Ni-Mn-Co-O inverse opal samples were prepared via a three step process as illustrated in Fig. 1a. Polystyrene sphere (PS) templates were infilled with an Ni-Mn-Co-O precursor solution and then annealed at 450 °C in air to crystallize the Ni-Mn-Co-O sample and to remove the sphere template. Interestingly, when the synthesis procedure was repeated without the PS template, nanoparticles (NPs) of Ni-Mn-Co-O were formed instead of an inverse opal network. The average diameters of these particles were quite small (~10 nm) as shown in Fig. 1b. A standard Ni-Mn-Co-O IO sample is shown in a scanning electron microscope (SEM) image in Fig. 1c. Ni-Mn-Co-O IO samples have a structure whereby islands of IO networks are formed instead of one continuous structure, as shown in Figure S1a–c. This is advantageous from an electrochemical point of view as it provides continuous transport paths for Li ions through the active phase (walls) and the electrolyte phase (pores)^22^. The cross-sectional thickness of a typical Ni-Mn-Co-O IO was ~19.8 μm, as shown in Figure S1d. After template infilling with the Ni-Mn-Co-O precursor solution, samples were allowed to dry initially at room temperature prior to annealing. In order to prepare carbon coated (C-coated) Ni-Mn-Co-O IO samples, a volume of a 0.1 M solution of ascorbic acid (Vitamin C) in IPA was dried on infilled sample before heating. An example of a C-coated Ni-Mn-Co-O IO sample is shown in Fig. 1d. It can be seen that there are some structural differences between the standard Ni-Mn-Co-O IO and the C-coated variant. The pores of the standard Ni-Mn-Co-O IO are circular due to the PS sphere template, however the pores of the C-coated Ni-Mn-Co-O IO appear hexagonal, with a honeycomb like structure. These features can be seen at different magnifications in the SEM images in Figure S2 and the transmission electron microscope (TEM) images in Figure S3.

TEM images of a standard Ni-Mn-Co-O IO sample are shown in Fig. 1e and f. The walls of the IO are comprised of an agglomeration of NPs as can be seen in Fig. 1e. These are the same NPs which are formed without the sphere template shown in Fig. 1b, the addition of the PS sphere template arranges the NPs into an IO architecture. The lattice fringes of the Ni-Mn-Co-O NPs have a spacing of ~0.446 nm, as shown in Fig. 1f. The electron diffraction pattern for an Ni-Mn-Co-O IO confirms the polycrystalline structure of the material, with several discrete rings visible in Fig. 1g. The electron diffraction pattern is a close match to several mixed transition metal oxide compounds, containing a combination of Ni, Mn and Co, namely cubic MnCo_2_O_4_, NiCo_2_O_4_ and NiMn_2_O_4_. The similarities in the crystal structure for Ni-Mn-Co-O IO samples and these compounds will be discussed in further detail in analysis of X-ray diffraction (XRD) patterns.

**Figure 2:**
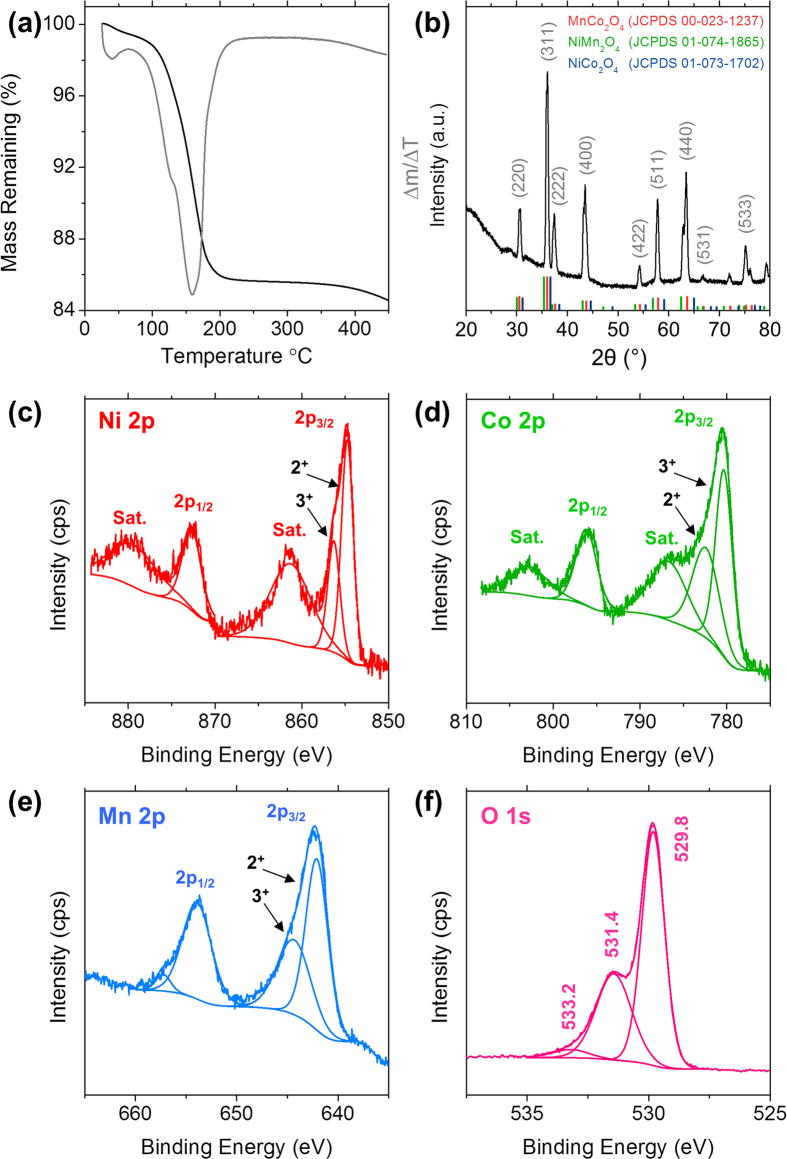
(**a**) Thermogravimetric analysis and derivative mass loss curves for Ni-Mn-Co-O precursor heated to 450 °C in air at a ramp rate of 5 °C/min. (**b**) XRD pattern of Ni-Mn-Co-O precursor after heating to 450 °C in air. XPS spectra of the (**c**) Ni 2p, (**d**) Co 2p, (**e**) Mn 2p and (**f**) O 1 s regions for a Ni-Mn-Co-O inverse opal.

Thermogravimetric analysis (TGA) of a dried Ni-Mn-Co-O precursor solution was performed in order to better appreciate the processes occurring during annealing, i.e. the removal of water and the crystallization of the Ni-Mn-Co-O sample. The mass loss curve presented in Fig. 2a indicates that ~15.5% mass is lost when heated to 450 °C. The derivative thermogravimetric curve shown in Fig. 2a consists of a weak peak at 40 °C a strong peak at 160 °C corresponding to mass losses of 1.0 and 9.6% respectively. The initial mass losses up to ∼100 °C are most likely due to the removal of physisorbed and chemisorbed water. The mass loss in the temperature range from 100–200 °C is due to the thermal decomposition of the Ni-Mn-Co-O precursors and the formation of a crystalline phase of Ni-Mn-Co-O. The majority of the mass was lost during heating to 200 °C, with only an additional ~1.4% mass being lost during heating from 200–450 °C. The XRD pattern for a standard Ni-Mn-Co-O IO is shown in Fig. 2b. Similar to the polycrystalline rings in the electron diffraction pattern in Fig. 1g, the reflections observed in the XRD pattern for Ni-Mn-Co-O can be readily indexed to cubic MnCo_2_O_4_ (JCPDS 00-023-1237) with an *Fd-3m* space group. We also note that the Ni-Mn-Co-O XRD pattern is also a close match for NiCo_2_O_4_ (JCPDS 01-073-1702) and NiMn_2_O_4_ (JCPDS 01-074-1865). A comparison between the Ni-Mn-Co-O XRD pattern and the reference patterns for all three mixed transition metal oxide compounds is shown in Fig. 2b. From XRD analysis the calculated lattice parameter for our Ni-Mn-Co-O IO is *a* = ~0.83 nm.

XPS analysis was used to probe the stoichiometry of an Ni-Mn-Co-O IO compared to that of binary mixed transition metal oxide compounds. XPS spectra of a standard Ni-Mn-Co-O IO sample displaying the Ni 2p, Co 2p, Mn 2p and O 1 s core-level photoemission are shown in Fig. 2c–f, respectively. Two main peaks can be seen in the Ni 2p spectrum at ~854.8 and 872.6 eV, corresponding to the Ni 2p_3/2_ and 2p_1/2_ levels respectively^19^. Both of these peaks contain shoulders and can be deconvoluted, indicating that they are composed of a mixture of Ni^2+^ and Ni^3+^, as shown in Fig. 2c 35. The two sub peaks associated with Ni^2+^ are located at ~854.8 and 872.3 eV, while the two associated with Ni^3+^ are located at ~856.4 and 874.0 eV. Two satellite peaks are also observed at ~880.0 and 861.4 eV. Comparison of the integrated areas of the peaks associated with each valence state indicates that ~34.1% of nickel present was in the Ni^3+^ valence state.

Two strong photoemission peaks were observed in the Co 2p spectrum at ~780.5 and 796.2 eV, corresponding to the Co 2p_3/2_ and 2p_1/2_ levels respectively^36^. Typically for Co_3_O_4_, with a mixed valence state of Co^2+^ and Co^3+^, the satellite peaks occur ~7–9 eV higher in binding energy than the main core-level signals^37^. Satellite peaks for the 2p_3/2_ and 2p_1/2_ levels were observed at ~786.9 and 802.9 eV, which is in close agreement with previous studies^38^,^39^,^40^. Due to the presence of the deconvoluted peaks present in the Co 2p_3/2_ and 2p_1/2_ levels and the satellite peaks, it is most likely that both Co^2+^ and Co^3+^ are present in the Ni-Mn-Co-O IO structure. Analysis of the deconvoluted peaks indicates that ~56.5% of the cobalt present was in the Co^3+^ valence state. The Mn 2p core level (Fig. 2e) consisted of two main peaks at ~642.3 and 653.9 eV, corresponding to the Mn 2p_3/2_ and 2p_1/2_ levels respectively^41^,^42^. Both peaks could be deconvoluted indicating the presence of Mn^2+^ and Mn^3+^. The two sub peaks at ~642.2 and 654.0 eV can be assigned to Mn^2+^, while the other two at ~644.5 and 657.3 eV are attributed to the presence of Mn^3+^ 43. Comparison of the integrated areas of the deconvoluted curves indicated that ~39.5% of the manganese present was in the Mn^3+^ valence state. XPS analysis confirms that all three elements are present on the surface of the Ni-Mn-Co-O IO material. The high resolution spectrum for the O 1 s shows three oxygen contributions. The peak at ~529.8 eV is typical of metal-oxygen bonds^44^,^45^. The peak at ~531.4 eV can be attributed to defects and a number of surface species including hydroxyls, chemisorbed oxygen or under-coordinated lattice oxygen^46^,^47^. The peak present at ~533.2 can be ascribed to multiplicity of physi- and chemisorbed water at or near the surface^44^,^45^. The quantification from high resolution spectra suggests that the surface stoichiometry of the Ni-Mn-Co-O IO is NiMn_1.7_Co_1.8_O_4_. The Ni-Mn-Co-O XRD pattern (Fig. 2b) was a close match with NiCo_2_O_4_ and NiMn_2_O_4_; both compounds are Ni deficient as is the case with our Ni-Mn-Co-O samples. Energy dispersive X-ray spectroscopy (EDS) and Raman spectroscopy were performed to confirm the graphitic nature of carbon coating of the ternary IO materials prepared with ascorbic acid, as shown in Figure S4. This data is discussed in detail in the Supplementary information.

In order to optimise our Ni-Mn-Co-O anode materials, the electrochemical performances of the various Ni-Mn-Co-O samples, shown in Fig. 1, were compared. Two variants of electrolyte were used in electrochemical testing, a standard 1 M solution of LiPF_6_ in EC/DMC with or without an electrolyte additive (3 wt.% vinylene carbonate (VC)). The addition of VC to the electrolyte has been reported to improve the stability of the solid electrolyte interface (SEI) layer and improve the capacity retention for anode materials such as Ge nanowires^48^,^49^,^50^, but its effectiveness in conversion-mode systems has received less attention. The experimental conditions for each Ni-Mn-Co-O sample can be summarised as follows: (i) Ni-Mn-Co-O nanoparticles and (ii) Ni-Mn-Co-O IO cycled in the standard electrolyte solution, (iii) Ni-Mn-Co-O IO and (iv) a C-coated Ni-Mn-Co-O IO cycled in the standard electrolyte solution containing 3 wt. % vinylene carbonate.

**Figure 3:**
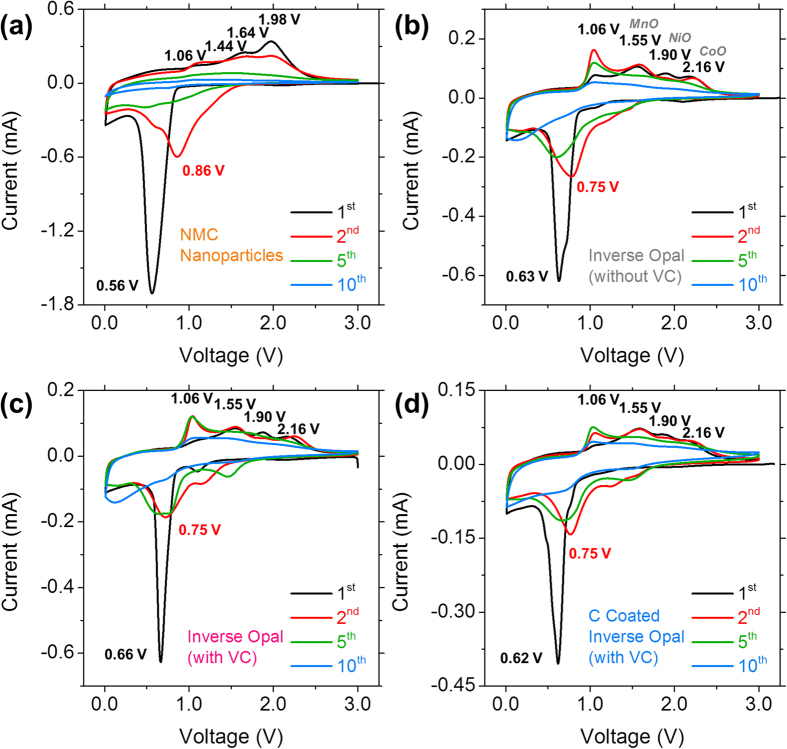
Cyclic voltammograms for (**a**) Ni-Mn-Co-O nanoparticles, Ni-Mn-Co-O IO cycled in electrolyte (**b**) without and (**c**) with a vinylene carbonate additive and (**d**) carbon-coated Ni-Mn-Co-O IO, acquired at a scan rate of 0.1 mV s^−1^.

A comparison of cyclic voltammograms (CVs) acquired for each Ni-Mn-Co-O sample is shown in Fig. 3. There is a sharp reduction peak in the first cathodic scan for Ni-Mn-Co-O nanoparticles at ~0.56 V, the potential of this peak increases to ~0.63 V for the Ni-Mn-Co-O IO, increases further to ~0.66 V for the Ni-Mn-Co-O IO cycled in the electrolyte containing VC and decreases slightly to ~0.62 V for the C-coated Ni-Mn-Co-O IO. This strong peak corresponds to the reduction of Ni-Mn-Co-O to Ni°, Mn° and Co° and the formation of amorphous Li_2_O and the SEI layer. The potential range in which the initial reduction occurs for all Ni-Mn-Co-O samples is in close agreement with potential values reported for other mixed transition metal oxide compounds, such as MnCo_2_O_4_ microspheres (~0.67 V)^20^, NiCo_2_O_4_ nanosheets (~0.67 V)^51^ and NiMn_2_O_4_ tremella like nanostructures (~0.60 V)^52^. The main reduction peak shifts to a higher potential of ~0.86 V in the second scan for the Ni-Mn-Co-O particles and ~0.75 V for all of the Ni-Mn-Co-O IO samples. Oxides of nickel, manganese and cobalt are known to behave as conversion mode materials (NiO, Mn_3_O_4_, Co_3_O_4_)^31^,^53^,^54^, i.e. during the first cathodic scan, each oxide is reduced to an unary metallic form. During the initial anodic scan Ni, Mn and Co are oxidised to reform oxides of each element. The resulting oxide nanoparticles can have diameters on the order of ~5 nm^55^,^56^. The shift in the potential for the reduction peak in the cathodic scan may be due to this decreased particle size^57^.

Four peaks were observed in the initial anodic scan for the Ni-Mn-Co-O nanoparticles at ~1.06, 1.44, 1.64 and 1.98 V respectively. For all other Ni-Mn-Co-O IO samples the four anodic peaks were observed at ~1.06, 1.55, 1.90 and 2.16 V. The anodic peaks at ~1.55, 1.90 and 2.16 V are associated with the formation of MnO, NiO and CoO respectively, and these processes are not hindered by the carbon-coating (Fig. 3(d))^20^,^52^. Thus, a single compositional Ni-Mn-Co-O ternary crystalline phase with IO structure re-oxidizes to three unary metal oxides from the corresponding reduced metals, in a single 3D open-worked honeycomb structure; Li reactions are electrochemically identified for each oxide from the first discharge. These peaks were shifted to slightly lower potentials for the Ni-Mn-Co-O NPs, possibly due to the reduced particle size and the disorder of the sample compared with the highly ordered IO samples. Interestingly the anodic peak at ~1.06 V was present at the same potential for Ni-Mn-Co-O NPs and all IO samples, and may be associated with the decomposition of Li_2_O formed during the initial cathodic scan.

Based on the analysis of the CV curves and previous reports^41^,^43^,^51^,^58^ and the surface stoichiometry suggested from XPS analysis, the Li reactions for Ni-Mn-Co-O IO materials are believed to proceed as follows:


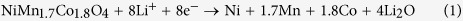



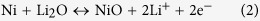



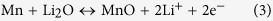



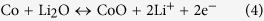


From Eqn. 1 the theoretical specific capacity for NiMn_1.7_Co_1.8_O_4_ is calculated to be ~665.5 mAh g^−1^. The theoretical capacity is lower than that of other transition metal oxides for example Co_3_O_4_ (~890 mAh g^−1^)^59^ due to molar mass of NiMn_1.7_Co_1.8_O_4_ being higher than that of Co_3_O_4_. It has previously been suggested for binary TMO compounds that, during anodic discharge, MnO and CoO may be further oxidized to Mn_3_O_4_ and Co_3_O_4_^19^,^52^, we appreciate that additional redox processes to those outlined in Eqns 1–4 may occur during cycling. Additionally, some regions in the core of the islands of IO (Figures S1 and S2) may take some time before they are initially reduced and that a number of cycles is required before stable state of three separate unary metal oxides is formed. This process would result in a reduction in anode charge capacity until it coulombically balances a stable discharge capacity (*vide infra*).

**Figure 4:**
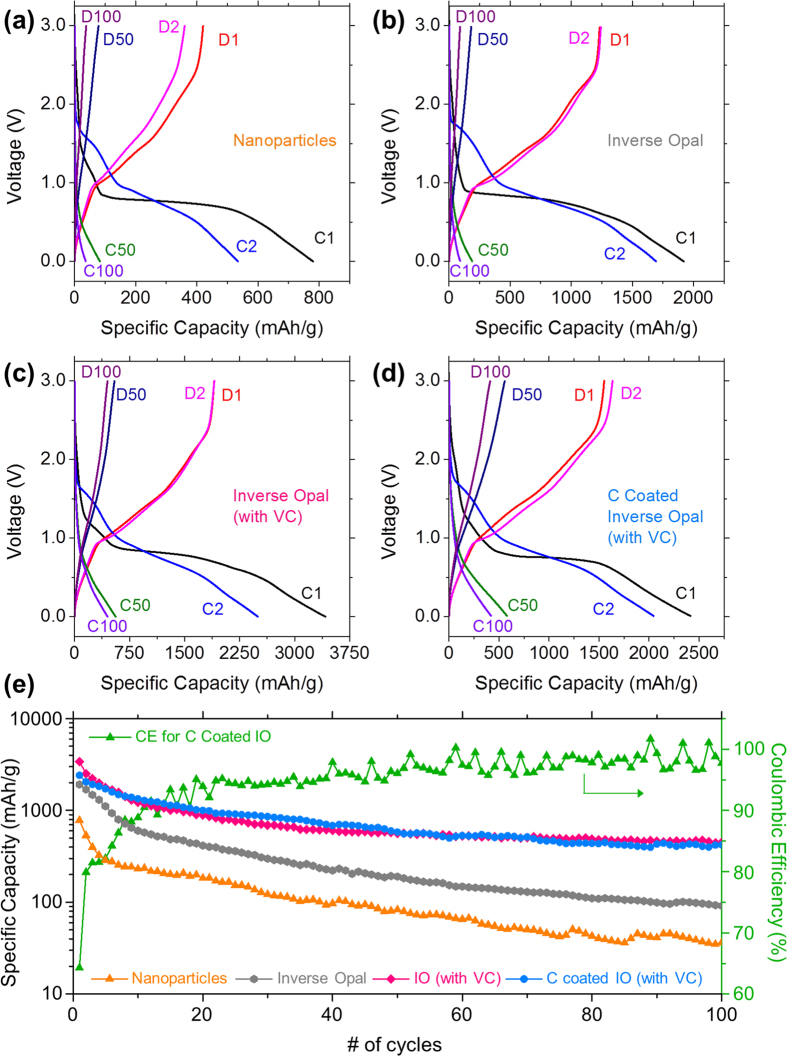
Charge and discharge voltage profiles for the 1^st^, 2^nd^, 50^th^ and 100^th^ cycle for (**a**) Ni-Mn-Co-O nanoparticles, Ni-Mn-Co-O IO cycled in electrolyte (**b**) without and (**c**) with a vinylene carbonate additive and (**d**) carbon coated Ni-Mn-Co-O IO, at a specific current of 150 mA g^−1^ in a potential window of 3.0–0.01 V. (**e**) Comparison of the specific capacity values obtained for all Ni-Mn-Co-O samples for 100 cycles.

Galvanostatic tests were performed to determine the effects of ordering and C-coating of Ni-Mn-Co-O samples on obtained specific capacity values and capacity retention. The galvanostatic charge and discharge curves for the 1^st^, 2^nd^, 50^th^ and 100^th^ cycles for each Ni-Mn-Co-O sample at a specific current of 150 mA g^−1^ are shown in Fig. 4. A long voltage plateau was observed in the initial charge curve for all Ni-Mn-Co-O samples between 0.9 and 0.7 V, corresponding to the reduction of Ni-Mn-Co-O to Ni^0^, Mn^0^ and Co^0^^58^,^60^. The potential range in which the plateau occurred is in close agreement with the potential at which the strong reduction peak occurred in the first cathodic CV scan for each sample, shown in Fig. 3. The initial charge capacity for the Ni-Mn-Co-O nanoparticles was ~780 mAh g^−1^, slightly higher than the theoretical capacity of 665 mAh g^−1^, this decreased to 535 mAh g^−1^ after the 2^nd^ charge. The irreversible capacity loss during the first charge may be attributed to the formation of an SEI layer^61^. From Fig. 4a it is clear that the disordered aggregation of Ni-Mn-Co-O nanoparticles suffer from severe capacity fading. However, introducing order via packing of the Ni-Mn-Co-O nanoparticles into an inverse opal structure increases the initial capacity and improves capacity retention. The initial specific capacity for an Ni-Mn-Co-O IO cycled in standard electrolyte was ~1920 mAh g^−1^, which is a significant increase over the Ni-Mn-Co-O NPs and nearly 3× the theoretical capacity for NiMn_1.7_Co_1.8_O_4_, however the capacity severely decayed after the 50^th^ and 100^th^ cycles as can be seen in Fig. 4b. The initial capacity increased to ~3420 mAh g^−1^ when the Ni-Mn-Co-O IO was cycled in electrolyte containing a VC additive and decreased to ~2415 mAh g^−1^ for the Ni-Mn-Co-O IO coated in carbon. The high initial capacities for all of the Ni-Mn-Co-O IO samples suggests a high degree of surface reactions (i.e. SEI formation, possible defect sites, lithium hydroxide/oxide species etc.)^16^,^62^,^63^,^64^. This is particularly pronounced for IOs due the high surface area/porosity as evidenced by the much lower initial specific capacities seen for the Ni-Mn-Co-O nanoparticles. After the second cycle the capacity for the C-coated Ni-Mn-Co-O IO was comparable to the non-C-coated IO, as shown in Fig. 4c and d.

A comparison of the specific capacity values obtained for each Ni-Mn-Co-O sample over 100 cycles plotted on a log scale is shown in Fig. 4e and on a linear scale in Figure S5. The disordered Ni-Mn-Co-O NPs suffer the most severe capacity fading with capacity values of 83 and 36 mAh g^−1^ after 50 and 100 cycles respectively. The capacity retention was improved by arranging the Ni-Mn-Co-O NPs into an inverse opal structure, obtaining capacity values of ~190 and 90 mAh g^−1^ after 50 and 100 cycles, respectively. The significant increase in the capacities obtained from the IO structured samples compared to immobilized powders of nanoparticles is due to the inherent properties of the 3D IO structure. The Ni-Mn-Co-O IO materials that cycle as three unary oxide of Ni, Mn and Co, possess a highly ordered, porous, interconnected structure with thin nanoparticulate walls. For conversion-mode materials, short Li^+^ diffusion distance matters during formation and decomposition of Li_2_O, and the IO system as an anode lower the decomposition overpotential at a voltage below that of conversion-mode cathode materials.

The addition of VC to the electrolyte significantly increased capacity values and improved capacity retention of the IO anodes. The trend in the capacity decay over the first ~10 cycles for the Ni-Mn-Co-O IO with and without VC are quite similar, with an initial large drop off followed by a far more gradual loss in capacity over the remaining cycles as the conversion to a triple unary metal oxide composition is stabilized during cycling. The capacity values for an Ni-Mn-Co-O IO cycled in electrolyte containing VC after the 50^th^ and 100^th^ cycles were 560 and 450 mAh g^−1^ respectively. The initial capacity of the C-coated Ni-Mn-Co-O samples cycled in electrolyte containing VC were lower than values obtained for the standard Ni-Mn-Co-O IO, however the capacities for the C coated IO were higher from the 10^th^ to the 50^th^ cycle as can be seen in Fig. 4e. The capacity values from the 50^th^ to the 100^th^ cycle for the C-coated Ni-Mn-Co-O IO sample were similar to the standard Ni-Mn-Co-O IO with values of ~580 and 420 mAh g^−1^ after the 50^th^ and 100^th^ cycles.

Although a significant difference in the capacity values obtained for the Ni-Mn-Co-O IO samples with or without carbon coating is not typically observed, as can be seen in Fig. 4e and Figure S5, the C-coating has an influence on the electrochemical response of the Ni-Mn-Co-O material. There are differences in the charge and discharge profiles for the Ni-Mn-Co-O IO electrodes with and without carbon coating can be seen in Fig. 4c and d. The first charge curve for the C-coated IO has a long flat plateau centred at ~0.75 V. The coulombic efficiency for the C-coated Ni-Mn-Co-O IO over 100 cycles is also shown in Fig. 4e. The initial coulombic efficiency is quite low (~65%) however after the 10^th^ cycle the efficiency remains >95%. While lower than commercially accepted efficiency, it is among the highest for conversion mode systems and improves with cycling as the three unary metal oxides undergo redox reaction simultaneously with a formation/decomposition of the lithium oxide species.

Capacity fading issues over initial cycles are well known for conversion mode materials, consequently the practice of preparing composites of conversion mode materials with graphene is quite common^65^. The significant change in the phase from a complex compound to the metal results in a large initial irreversible capacity loss. A previous report on the electrochemical performance of Co_3_O_4_ nanoparticles found severe capacity fading when cycled on their own and the preparation of a composite with graphene sheets significantly enhanced performance^37^. However graphene actively stores charge when cycled in a potential window from 3.0–0.01 V (vs Li/Li^+^) and we desired to investigate the fundamental electrochemical response of our Ni-Mn-Co-O material in the absence of any materials which actively contribute towards the total stored charge.

With increased cycling the Ni-Mn-Co-O samples likely becomes a matrix of NiO, MnO and CoO, as explained by Eqns 1–4. It may take some time by successive cycling before all of the Ni-Mn-Co-O material matrix undergoes the initial conversion mode reactions (including reduction and re-oxidation to consistent oxidation states). We propose this as a reason why the coulombic efficiency improves after the first 10 cycles and remains very consistent thereafter - the efficiency of the charge and discharge curves together are always similar, yet both increase with successive cycling. SEM images of a C-Coated Ni-Mn-Co-O sample after 100 cycles at a specific current of 150 mA g^−1^ are shown in Figure S7. Due to the destructive nature of the conversion mode reaction the IO samples are converted to an agglomeration of nanoparticles after cycling. However, nanoparticles without 3D structuring are significantly less capable of extended cycling at similar rates. The capacity values obtained for the Ni-Mn-Co-O IO and C-coated Ni-Mn-Co-O IO samples cycled in electrolyte containing VC are higher than previously reported values for other mixed transition metal oxide anode materials such as NiMn_2_O_4_ and MnCo_2_O_4_^20^,^41^,^60^,^66^,^67^. A detailed comparison of the capacities obtained for our C-coated Ni-Mn-Co-O IO to representative literature values is present in Table S2. Further to this, the capacity values obtained for our Ni-Mn-Co-O IO anode materials after 100 cycles were >400 mAh g^−1^, consequently they are more than suitable for pairing in a full cell configuration with the majority of cathode materials reported for higher energy density cells^68^,^69^. The electrochemical performance of C-coated Ni-Mn-Co-O IO samples was further investigated by galvanostatic cycling using series of different specific currents ranging from 75–450 mA g^−1^ and rate capability testing as shown in Figure S8. These tests are discussed in detail in the Supplementary information.

In order to confirm that our Ni-Mn-Co-O IOs are a true anode material unlike the classic lithiated NMC cathode material (LiNi_0.33_Mn_0.33_Co_0.33_O_2_), Ni-Mn-Co-O IOs were cycled galvanostatically in a commonly used cathode potential window for Li-NMC based cathode materials (4.4–2.5 V)^32^. The resulting discharge and charge curves are shown in Figure S9. The initial discharge capacity for an Ni-Mn-Co-O IO sample cycled in a cathode potential window was ~6.3 mAh g^−1^. The poor electrochemical performance of the Ni-Mn-Co-O IOs when cycled in a cathode potential window compared to the impressive capacities obtained when cycled in an anode potential window confirm that our Ni-Mn-Co-O IO samples are an anode material. Ni-Mn-Co-O IO samples are ternary analogs similar to other well-known binary TMO anode materials such as MnCo_2_O_4_ and NiCo_2_O_4_ 70,71.

**Figure 5:**
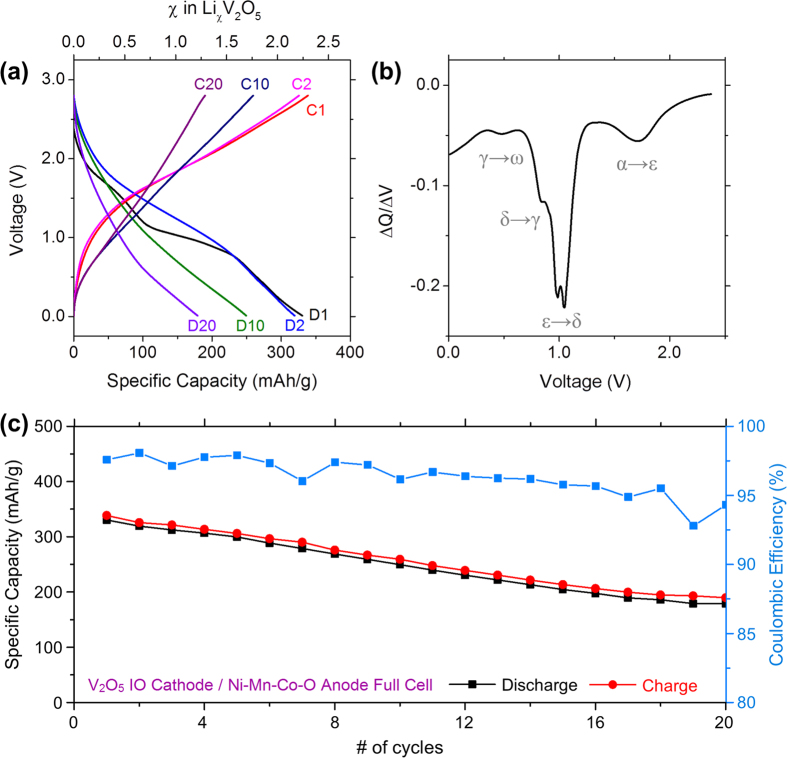
(**a**) Charge and discharge voltage profiles for the 1^st^, 2^nd^, 10^th^ and 20^th^ cycles for a full Li-ion cell consisting of a V_2_O_5_ IO cathode and a pre-charged Ni-Mn-Co-O IO anode. (**b**) Differential charge curve for the first discharge demonstrating the different phases associated with the intercalation of Li^+^ within vanadium oxide (**c**) The discharge and charge capacities and the Coulombic efficiency obtained over 20 cycles for the V_2_O_5_ IO/Ni-Mn-Co-O IO full cell.

C-coated Ni-Mn-Co-O IOs demonstrated impressive capacities when galvanostatically cycled in a half cell configuration versus a pure Li counter electrode as shown in Fig. 4 and S5. We have previously reported a full cell consisting of a V_2_O_5_ IO cathode paired with an electrochemically prelithiated Co_3_O_4_ IO anode^31^. In order to investigate the practical potential of C-coated Ni-Mn-Co-O IO anodes, they were tested in a full cell arrangement against a V_2_O_5_ IO cathode. Similar to our previous work, the Ni-Mn-Co-O IO anode was electrochemically pre-charged by a single charge against a Li metal counter electrode before being paired with a V_2_O_5_ IO cathode. The electrochemical characterization of the V_2_O_5_ cathode/Ni-Mn-Co-O anode IO Li-ion battery is presented in Fig. 5. The OCV for V_2_O_5_ IOs vs Li metal is typically ~3.6 V (vs. Li/Li^+^)^29^,^31^, whereas for the V_2_O_5_ IO/Ni-Mn-Co-O IO full cell (in a 2-electrode configuration, without a dedicated Li reference electrode) the OCV was ~2.4 V, approximately 1.2 V lower. Also it is worth nothing that the potential values stated for the V_2_O_5_ IO/Ni-Mn-Co-O IO full cell are not stated vs. Li/Li^+^, instead they are with respect to the pre-charged Ni-Mn-Co-O IO anode. V_2_O_5_ is typically cycled in a potential window ranging from 4.0–1.2 V (vs. Li/Li^+^) when cycled in a half cell arrangement^72^,^73^. However due to the OCV decreasing by 1.2 V in the full cell, the effective potential window for our full cell was 2.8–0.01 V.

The voltage profiles for the 1^st^, 2^nd^, 10^th^ and 20^th^ cycles are shown in Fig. 5a. The well-known phases of Li^+^ intercalation within vanadium oxide were observed in the first discharge curve and can also be seen in the differential charge curve for the first discharge in Fig. 5b. Due to the initial lower OCV of the full cell compared to a V_2_O_5_ half-cell, each of the Li^+^ intercalation phases are also shifted to lower potentials. After the first and second discharges ~2.24 and 2.18 mol of Li were intercalated per V_2_O_5_ unit, this value decreased to ~1.70 and 1.21 mol after the 10^th^ and 20^th^ discharges respectively. During the initial charging of the Ni-Mn-Co-O IO anode and prior to full cell assembly, Ni-Mn-Co-O is reduced and Li_2_O is formed, as described by Eqn. 1. Consequently, the Li^+^ source in the V_2_O_5_ IO/Ni-Mn-Co-O IO full cell is Li_2_O. The amount of Li intercalated per V_2_O_5_ unit over the first 20 cycles indicates that Li_2_O can be reversibly reduced to Li^+^ and is an effective Li^+^ source for the V_2_O_5_ IO/Ni-Mn-Co-O IO full cell.

The specific capacity values obtained over the first 20 cycles and the Coulombic efficiency are shown in Fig. 5c. The specific capacity is stated in terms of the mass of the cathode, i.e. the V_2_O_5_ IO. The initial discharge capacity for the V_2_O_5_ IO/Ni-Mn-Co-O IO full cell was ~330 mAh g^−1^ which is slightly higher than the initial capacity we previously reported for the V_2_O_5_ IO/Co_3_O_4_ IO full cell (~290 mAh g^−1^). The specific capacity decreased slightly to ~320 mAh g^−1^ after the second discharge and retained a value of ~250 and 180 mAh g^−1^ after the 10^th^ and 20^th^ discharges, respectively. The capacity values obtained the V_2_O_5_ IO/Ni-Mn-Co-O IO full cell are greater than previously reported values for nanostructured V_2_O_5_ half cells^5^,^74^. The electrochemical performance of the V_2_O_5_ IO/Ni-Mn-Co-O IO full cell again demonstrates that our Ni-Mn-Co-O IOs behave as an anode material which can be cycled reversibly in a full cell configuration when paired with a V_2_O_5_ cathode.

There are numerous reports investigating lithiated NMC compounds as cathode materials for Li-ion batteries. This report represents the first systematic investigation into the application of an Ni-Mn-Co-O compound as a conversion mode anode material with an open-worked structural network that helps to improve mass transport of species. Various morphologies of Ni-Mn-Co-O were examined and compared including nanoparticles, inverse opals and C-coated inverse opals. In order to obtain high capacity via conversion mode reactions, a single crystalline honeycombed IO structure of Ni-Mn-Co-O material was first formed, that converts via reversible redox reactions and Li_2_O formation to a 3D structured assembly of three (MnO, CoO and NiO) oxides, that facilitates efficient reactions with Li. A carbon coating maintains the structure without clogging the open-worked IO pore structure for electrolyte penetration and mass transport of products. Electron diffraction and XRD analysis confirmed that Ni, Mn and Co were all present within the crystal structure of the various Ni-Mn-Co-O samples. XPS analysis indicated a surface stoichiometry of the Ni-Mn-Co-O IO samples of NiMn_1.7_Co_1.8_O_4_.

The electrochemical performance of Ni-Mn-Co-O NPs was improved by engineering the nanoparticles into a highly porous IO architecture. By introducing this order, the Ni-Mn-Co-O IOs showed significantly increased capacity values compared to the basic constituent nanoparticles. This report highlights the scope for tailoring the morphology of mixed transition metal oxide conversion mode materials to improve electrochemical performance. The capacity values for Ni-Mn-Co-O IOs were significantly increased by incorporating a vinylene carbonate additive to the electrolyte, with capacity retention also substantially improved. Further optimization of the Ni-Mn-Co-O IO samples was investigated by carbon coating via the addition of a solution of ascorbic acid prior to annealing. C-coated Ni-Mn-Co-O IOs demonstrated promising high-rate performance and high capacity values, which were greater than the theoretical capacity for NiMn_1.7_Co_1.8_O_4_ even after 50 cycles. C-coated Ni-Mn-Co-O IO anodes were also successfully paired with a V_2_O_5_ IO cathode to form a full Li-ion cell. The specific capacities obtained for the V_2_O_5_ IO/Ni-Mn-Co-O IO full cell are greater than previously reported values for nanostructured V_2_O_5_ half cells. This demonstrates that the C-coated Ni-Mn-Co-O IOs in the form of multi oxide conversion mode anodes are more effective in improving round trip energy efficiency per cycle.

## Methods

### Inverse opal anode material formation

The Ni-Mn-Co-O IO was prepared by a two-step process. Firstly, a solution of polystyrene (PS) spheres (Polysciences Inc., diameter ~500 nm) in isopropanol was drop cast on to 1 cm^2^ pieces of stainless steel; the sphere templates were then infilled with a 1:1:1 (v/v/v) mixture of a 0.1 M solution of NiCl_2_.6H_2_O, MnCl_2_.4H_2_O and CoCl_2_ in isopropyl alcohol (IPA). Secondly, the infilled spheres where heated at 450 °C in air for 12 h, to remove the sphere templates and to crystallize the material. Ni-Mn-Co-O nanoparticles were also prepared using the same method but without a PS sphere template. Carbon-coated Ni-Mn-Co-O IOs were prepared by dropping a volume of a 0.1 M solution of ascorbic acid (Vitamin C) in IPA onto an infilled Ni-Mn-Co-O sample before annealing to 450 °C.

### Material characterization

TEM analysis was conducted using a JEOL JEM-2100 TEM operating at 200 kV. SEM analysis was performed using an FEI Quanta 650 FEG high resolution SEM at an accelerating voltage of 10 kV. TGA was performed using a Mettler Toledo TGA/DSC1. Ni-Mn-Co-O samples were placed in an alumina crucible and heated to 450 °C in air at a heating rate of 5 °C min^−1^. XRD analysis was performed using a Phillips Xpert PW3719 diffractometer using Cu Kα radiation. (Cu Kα, λ = 0.15418 nm, operation voltage 40 kV, current 40 mA). XPS spectra were acquired on an Oxford Applied Research Escabase XPS system equipped with a CLASS VM 100 mm mean radius hemispherical electron energy analyzer with multichannel detectors in an analysis chamber with a base pressure of 5.0 × 10^–10^ mbar. Survey scans were recorded between 0 and 1400 eV with a step size of 0.7 eV, dwell time of 0.5 s, and pass energy of 100 eV. Core level scans were acquired with a step size of 0.1 eV, dwell time of 0.5 s, and pass energy of 20 eV averaged over 10 scans. A non-monochromated Al Kα X-ray source at 200 W power was used for all scans. All spectra were acquired at a take-off angle of 90° with respect to the analyzer axis and were charge corrected with respect to the C 1 s photoelectric line. Data was processed using CasaXPS software where a Shirley background correction was employed and peaks were fitted to Voigt profiles.

### Electrochemical characterization

All electrochemical results presented in this report were performed using a BioLogic VSP Potentiostat/Galvanostat. The electrochemical properties of Ni-Mn-Co-O samples were investigated in a half cell configuration against a pure Li counter electrode in a two electrode, stainless steel split cell (a coin cell assembly that can be disassembled for post-mortem analysis). The electrolytes used consisted of a 1 mol dm^−3^ solution of lithium hexafluorophosphate salts in a 1:1 (v/v) mixture of ethylene carbonate in dimethyl carbonate with or without 3 wt% vinylene carbonate. The separator used in all split cell tests was a glass fiber separator (El-Cell ECC1-01-0012-A/L, 18 mm diameter, 0.65 mm thickness). The mass loading for all Ni-Mn-Co-O samples was ~0.5–1.0 mg, no additional conductive additives or binders were added. Cyclic voltammetry was performed using a scan rate of 0.1 mV s^−1^ in a potential window of 3.0–0.01 V. Galvanostatic cycling was performed using a range of specific currents (75–450 mA g^−1^) in a potential window of 3.0–0.01 V. For testing in a full cell arrangement against a V_2_O_5_ IO cathode, the C-coated Ni-Mn-Co-O IO anode was electrochemically pre-charged by a single charge against a Li metal counter electrode. V_2_O_5_ IOs were prepared using the same method we have previously reported^31^. V_2_O_5_ IO/Ni-Mn-Co-O IO full cells were cycled in a potential window of 3.0–0.2 V in a 2-electrode configuration.

## Additional Information

**How to cite this article:** McNulty, D. *et al*. Carbon-Coated Honeycomb Ni-Mn-Co-O Inverse Opal: A High Capacity Ternary Transition Metal Oxide Anode for Li-ion Batteries. *Sci. Rep.* **7**, 42263; doi: 10.1038/srep42263 (2017).

**Publisher's note:** Springer Nature remains neutral with regard to jurisdictional claims in published maps and institutional affiliations.

## Supplementary Material

Supplementary Information
