# Supplementary material for: Carbon-Coated Honeycomb Ni-Mn-Co-O Inverse Opal: A High Capacity Ternary Transition Metal Oxide Anode for Li-ion Batteries
Source: Sci Rep. 2017 Feb 10;7:42263. doi: 10.1038/srep42263 (PMC5301490; doi:10.1038/srep42263)
Supplement: Supplementary Information [file srep42263-s1.pdf]

Supplementary Information for:

Carbon-Coated Honeycomb Ni-Mn-Co-O Inverse Opal: A High Capacity Ternary Transition Metal Oxide Anode for Li-ion Batteries

David McNulty, Hugh Geaney and Colm O'Dwyer*

Department of Chemistry, University College Cork, Cork T12 YN60, Ireland

Micro-Nano Systems Centre, Tyndall National Institute, Lee Maltings, Cork T12 R5CP, Ireland

* Corresponding Author: c.odwyer@ucc.ie

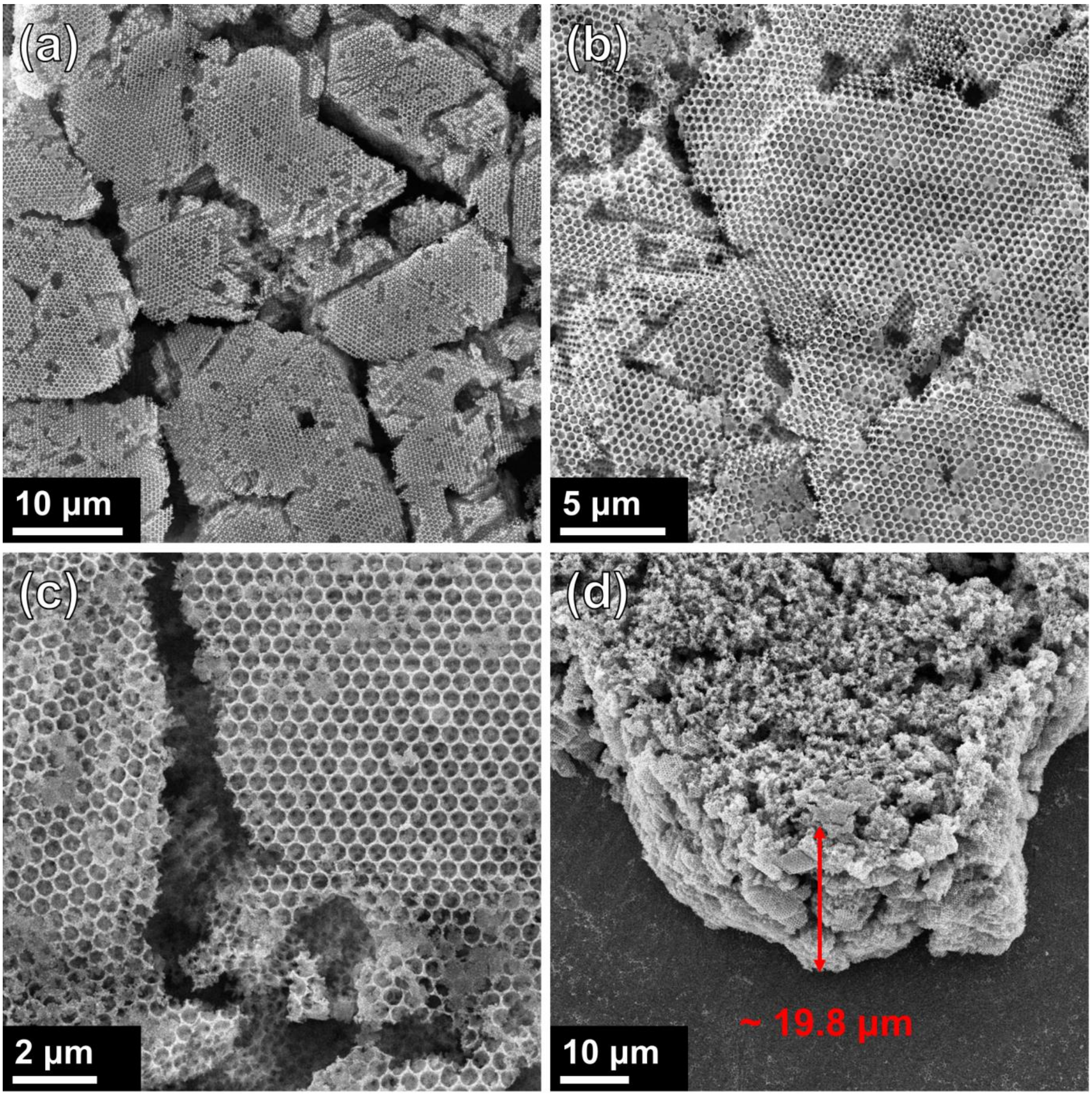

Figure S1. SEM images showing (a)-(c) the bicontinuous structure of an Ni-Mn-Co-O IO sample, (d) tilt corrected SEM image showing the thickness of a typical Ni-Mn-Co-O IO.

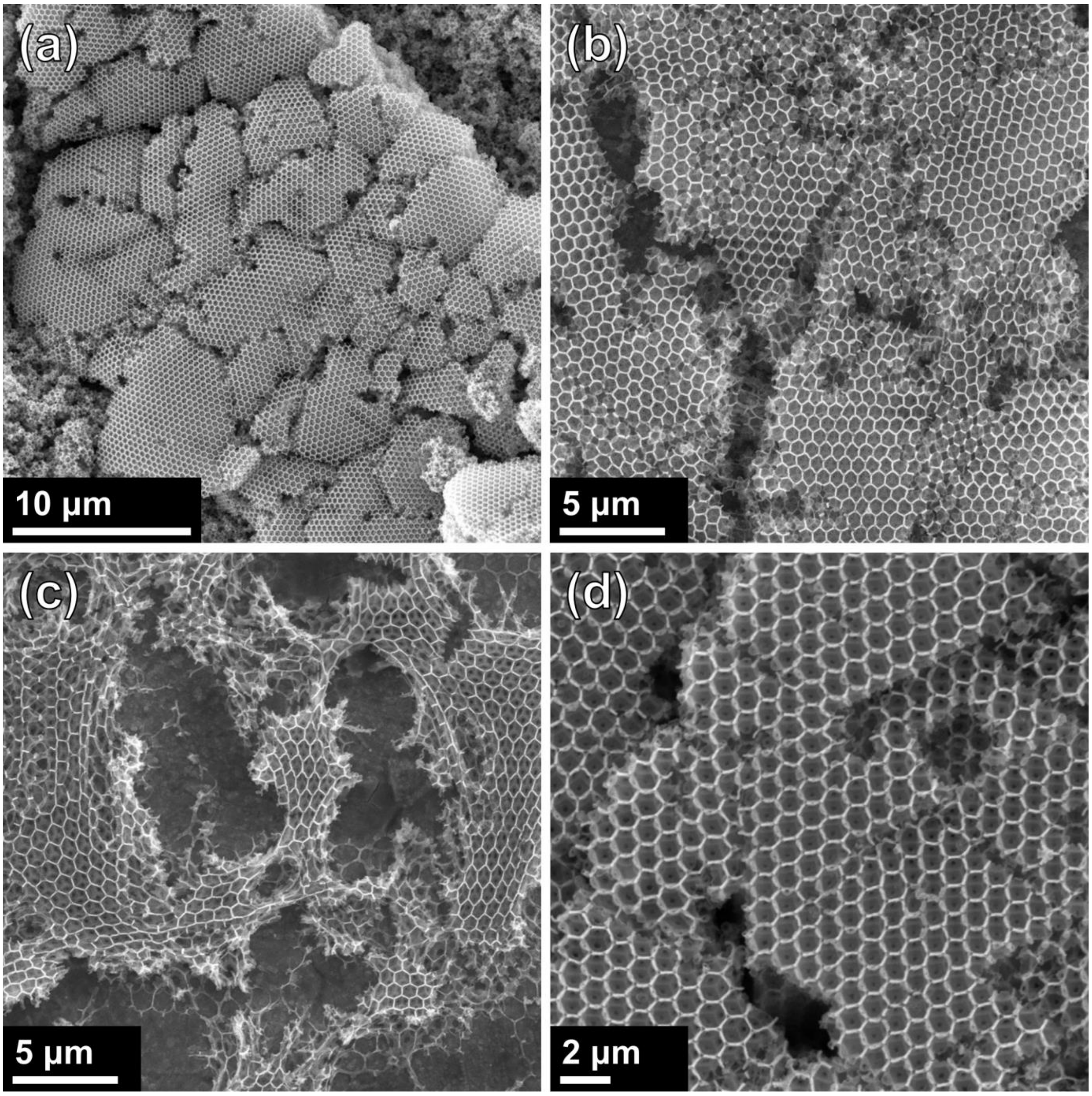

Figure S2. SEM images of different magnifications of typical C-coated Ni-Mn-Co-O IO samples.

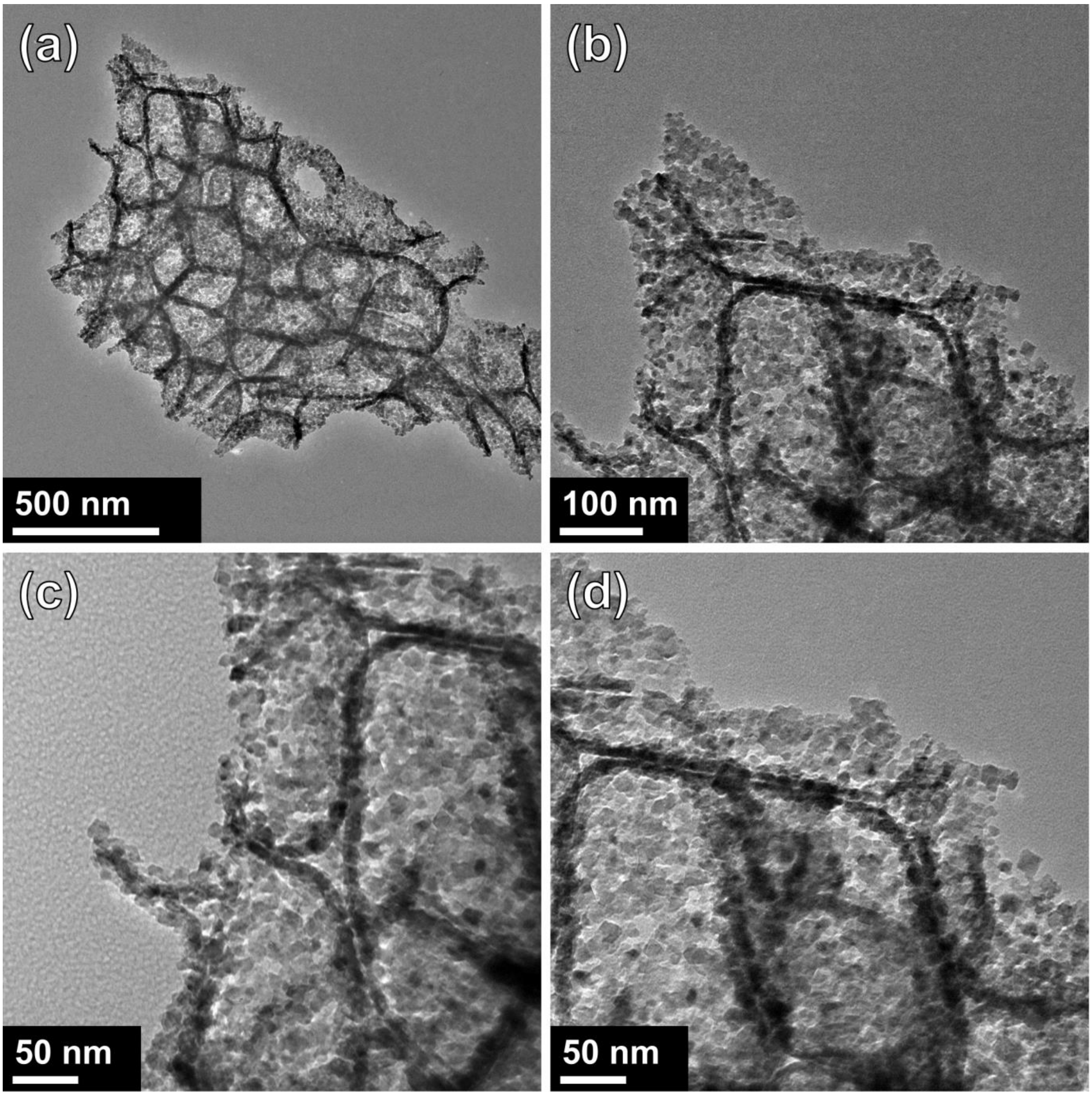

Figure S3. TEM images of different magnifications of typical C-coated Ni-Mn-Co-O IO samples.

High magnification SEM images of a standard Ni-Mn-Co-O IO and a C coated Ni-Mn-Co-O IO are shown in Figure S4a and b respectively. The effect of the addition of ascorbic acid on the walls of the IO can be seen clearly. Without ascorbic acid the pores of the IO are circular and the walls are smooth whereas with ascorbic acid the IO walls are distorted and appear to have a rougher surface and the pores become hexagonal. EDS spectra were acquired over the areas represented in the SEM images and a comparison of the spectra acquired for both samples is shown in Figure S4c. The spectra for both samples are quite similar with both confirming the presence of Ni, Mn, Co and O within the IO structures. The characteristic C peak at ~ 0.27 keV is present in both materials, however the intensity of the peak is higher for the C coated Ni-Mn-Co-O IO sample. The atomic percentages (at.%) of each element present are listed in Table S1. There was ~ 7.59 % C in the standard Ni-Mn-Co-O IO sample, with is most likely due to adventitious carbon on the surface of the IO. The at.% of C for the Ni-Mn-Co-O IO samples prepared with ascorbic acid almost doubled to ~ 14.11 %. For the standard Ni-Mn-Co-O IO the ratio of C:Ni is ~ 1:1, however for the carbon coated sample the ratio is ~ 2.5:1. EDS analysis confirms a substantial increase in C content for the IOs prepared with ascorbic acid compared to those prepared without, and it conformally coats the surface of the IO material.

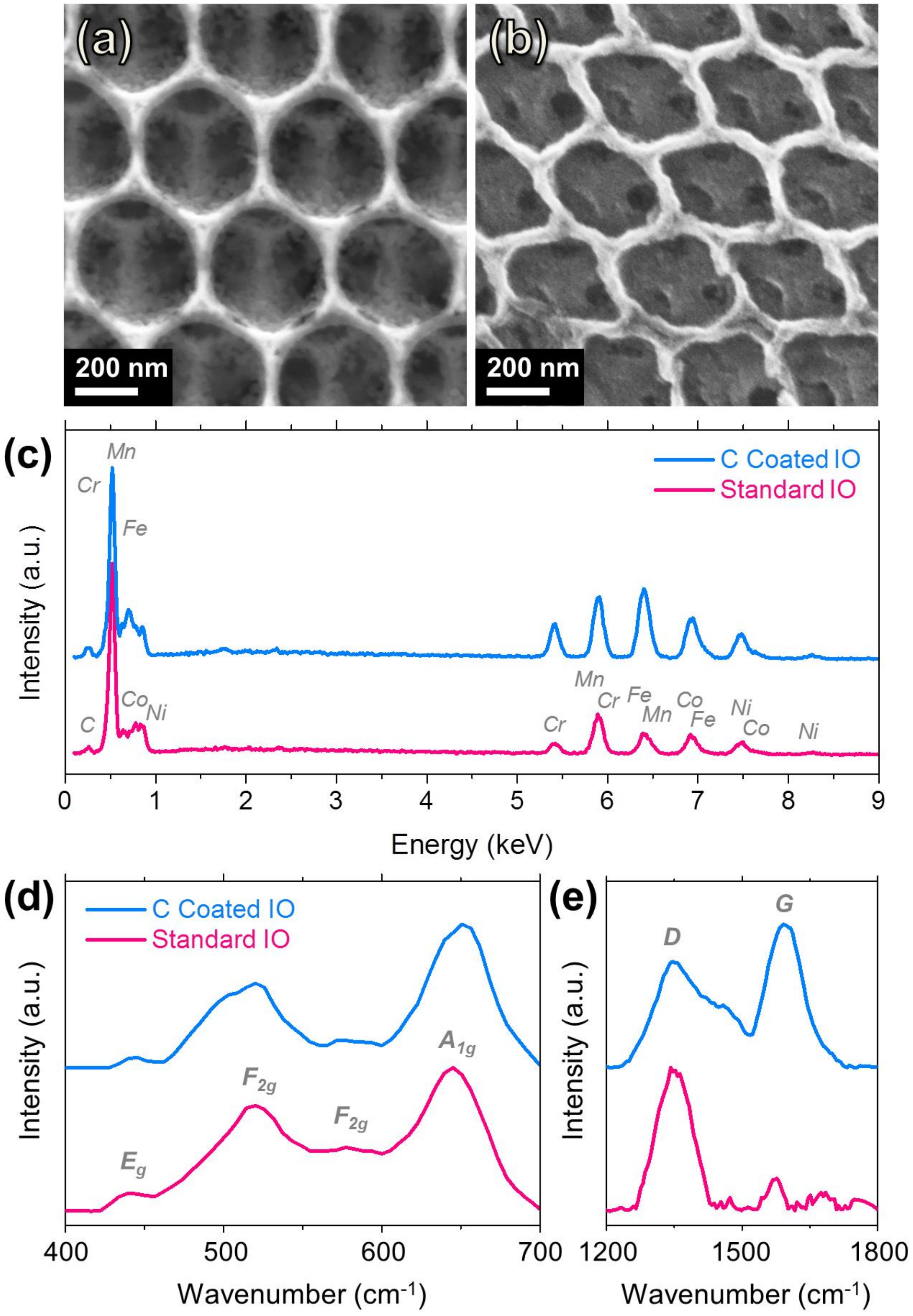

Figure S4. High magnification SEM images of (a) a standard Ni-Mn-Co-O IO and (b) a C coated Ni-Mn-Co-O IO. (c) EDS spectra for a standard Ni-Mn-Co-O IO and a C coated Ni-Mn-Co-O IO acquired from the areas represented in the SEM images. Raman spectra for a standard Ni-Mn-Co-O IO and a C coated Ni-Mn-Co-O IO from (d) 400 – 700 cm^{-1} and © 1200 – 1800 cm^{-1}.

A comparison of the Raman spectra for a standard Ni-Mn-Co-O IO and a C-coated Ni-Mn-Co-O IO is shown in Figure S4d. The Raman spectrum for the as prepared Ni-Mn-Co-O IO contains four distinct bands at ~ 440, 519, 575 and 644 cm^{-1}. These bands are in close agreement with previously reported values for binary TMO compounds such as $MnCo_2O_4$ and $NiCo_2O_4$ and correspond to the E_g, F^2_{2g}, F^3_{2g} and A_{1g} modes, respectively.[1,2] These bands are all slightly shifted for the C-coated Ni-Mn-Co-O IO sample to ~ 442, 520, 579 and 648, respectively, but predominantly show that the nature of the Ni-Mn-Co-O nanocrystalline component of the IO material remain unchanged when coated with carbon. Raman scattering date in Figure S4e shows that the C-coating exhibits the characteristic D-band at ~1350 cm^{-1}, associated with disordered carbon [3,4] for both Ni-Mn-Co-O IO samples. A very weak G-band was observed in the Raman spectrum for the standard Ni-Mn-Co-O IO, however the G-band dominates this region of the Raman spectrum for the C-coated Ni-Mn-Co-O IO. This confirms that there is significantly more C present for the C-coated Ni-Mn-Co-O IO and that it structure is similar to a layered graphitic-type carbon that coats the surface of the Ni-Mn-Co-O IO anode.

	Atomic %	
Element	Standard IO	C Coated IO
C	7.59	14.11
Ni	7.46	5.52
Mn	11.87	7.52
Co	9.31	7.53
O	53.98	51.37
Cr*	2.63	3.5
Fe*	7.16	10.45

Table S1. Atomic % of the various elements present in the Ni-Mn-Co-O IO samples prepared on a stainless steel substrate. * The presence of Cr and Fe in the EDS spectra is due to the stainless steel substrate on which the IO samples are prepared.

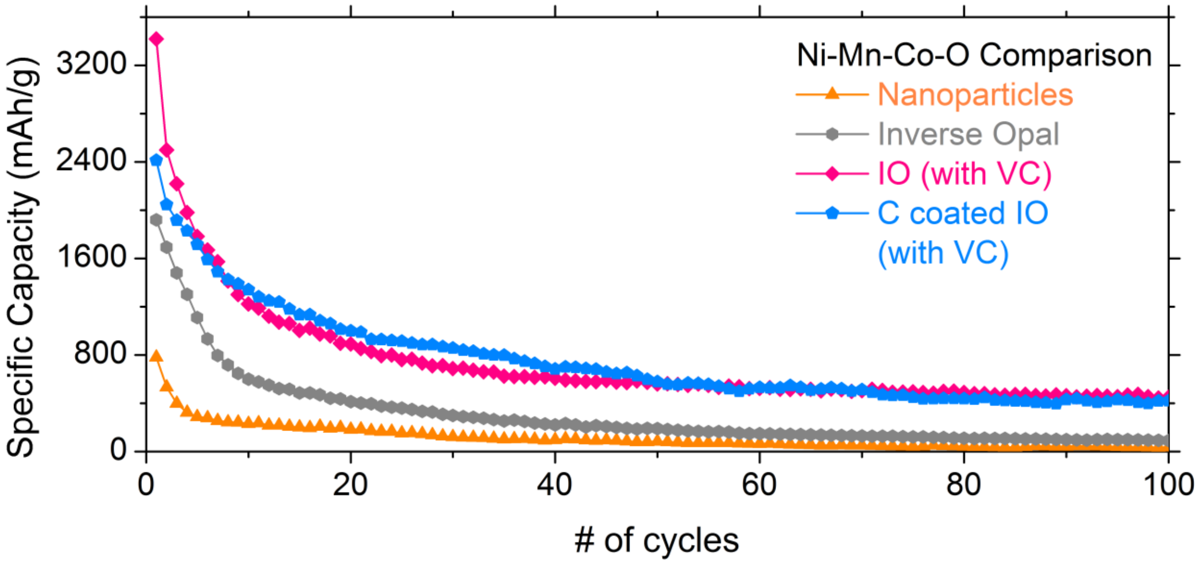

Figure S5. Comparison of the specific capacity values, plotted on a linear scale, obtained for all Ni-Mn-Co-O samples for 100 cycles.

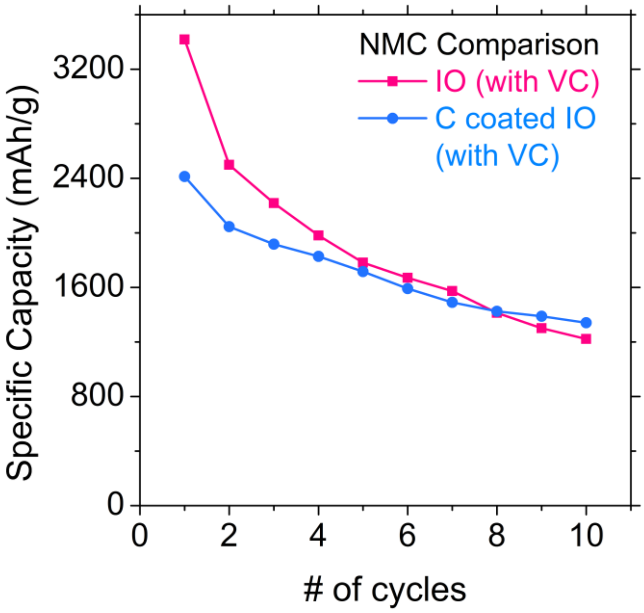

Figure S6. Comparison of the specific capacity values obtained for Ni-Mn-Co-O IO samples with and without C-coating over 10 cycles.

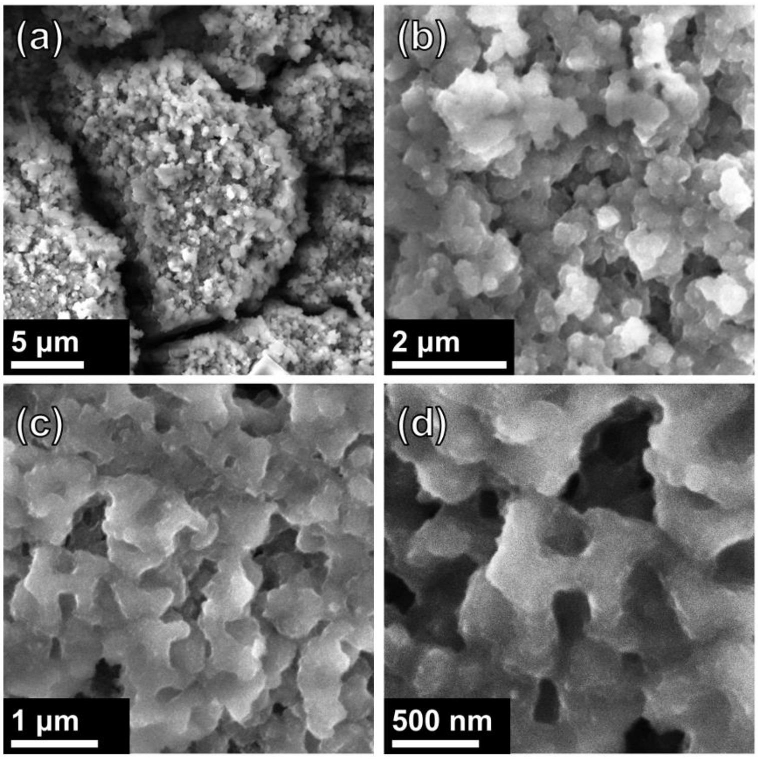

Figure S7. SEM images of a C-Coated Ni-Mn-Co-O sample after 100 cycles at a specific current of 150 Ma g^{-1}

The electrochemical performance of C-coated Ni-Mn-Co-O IO samples was further investigated by galvanostatic cycling using series of different specific currents ranging from 75 – 450 mAh g^{-1}. The resulting specific capacity values are compared in Figure S8a. The highest capacity values were obtained at the lowest specific currents, achieving capacity values of ~ 970 and 675 mAh g^{-1} after the 25th and 50th cycles. These values decreased to 915 and 580 mAh g^{-1}, when cycled at 150 mAh g^{-1}. Increasing the specific current from 75 to 150 mAh g^{-1} resulted in slightly decreased capacity values, however increasing the specific current further to 300 Ma g^{-1} significantly decreased the capacity values obtained to ~ 640 and 430 mAh g^{-1} after the 25th and 50th cycles, respectively. These values decreased further to 605 and 395 mAh g^{-1} when the specific current was increased to 450 mAh g^{-1}. Capacity values greater than the theoretical values of the most commonly used anode materials ($Li_4Ti_5O_{12}$ = 175 mAh g^{-1}, graphite = 372 mAh g^{-1}) were obtained even when cycled at a high

specific current (450 Ma g^{-1}). Achieving significant capacity values at high specific current is crucial for practical batteries where fast discharge and charge is required, hence the capacity values obtained for C-coated Ni-Mn-Co-O IOs are quite substantial. At the lowest specific current, capacity values greater than the theoretical capacity for $NiMn_{1.7}Co_{1.8}O_4$ (~ 665.5 mAh g^{-1}) were obtained over 50 cycles.

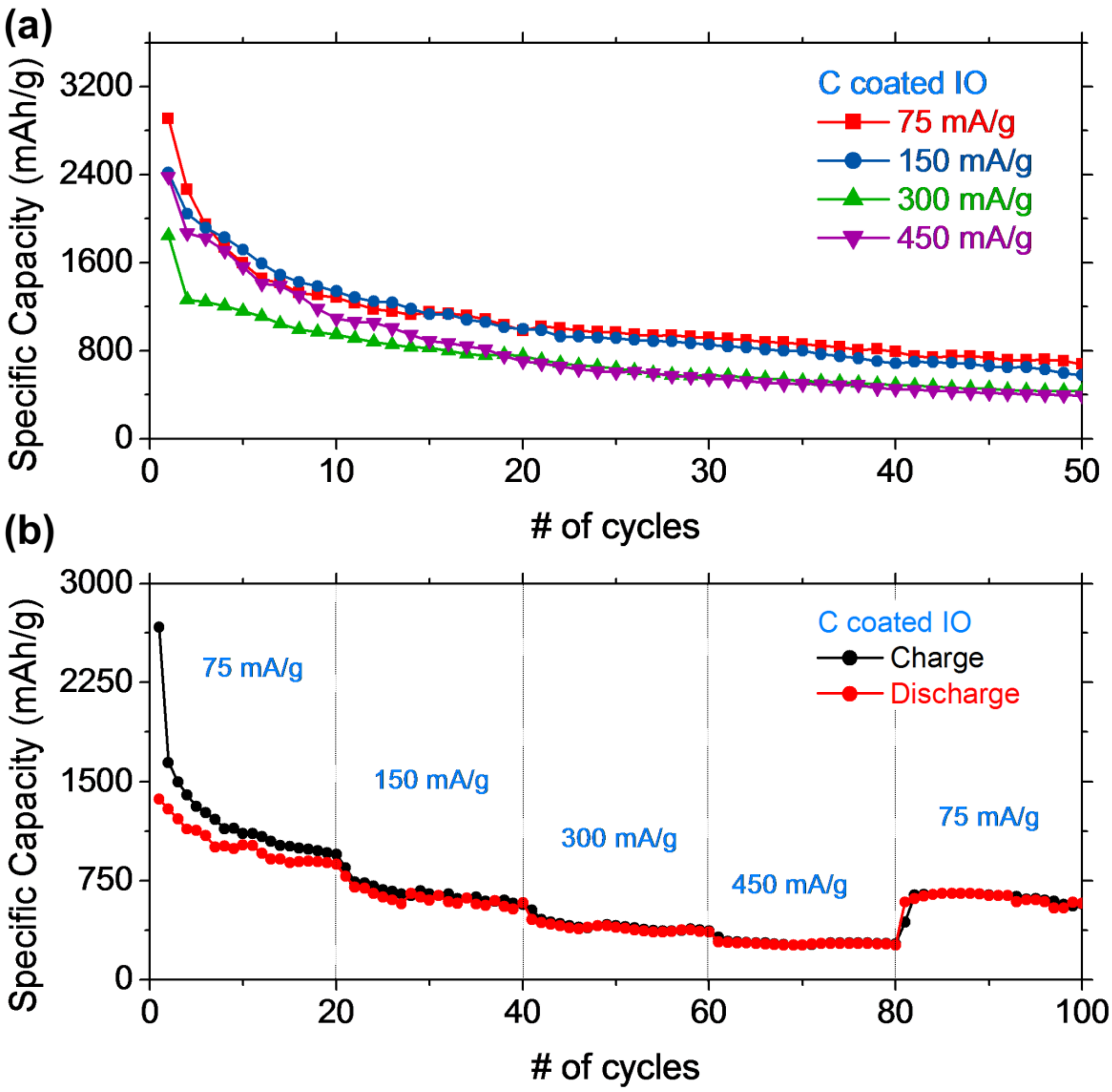

Figure S8. (a) Charge and discharge voltage profiles for the 1st, 2nd, 50th and 100th cycle for (a) Ni-Mn-Co-O nanoparticles, Ni-Mn-Co-O IO cycled in electrolyte (b) without and (c) with a vinylene carbonate additive and (d) carbon coated Ni-Mn-Co-O IO, at a specific current of 150 Ma g^{-1} in a potential window of 3.0 – 0.01 V. € Comparison of the specific capacity values obtained for C-coated Ni-Mn-Co-O IO samples cycled using specific currents of 75, 150, 300 and 450 Ma g^{-1}, (b) Rate capability test for C-coated Ni-Mn-Co-O IO over 100 cycles, using specific currents ranging from 75 – 450 Ma g^{-1}.

The rate performance of C-coated Ni-Mn-Co-O IO samples was investigated to determine the capacity recoverability when cycled at high specific currents, as shown in Figure S8b. C-coated Ni-Mn-Co-O IOs were cycled at various specific currents (75 – 450 mAh g^{-1}). C-coated Ni-Mn-Co-O IOs demonstrated excellent rate capability with an average charge capacity of 1225, 650, 405 and 280 mAh g^{-1} when the specific current increased every twenty cycles from 75 to 150, 300 and 450 Ma g^{-1}. Upon returning to the initial specific current of 75 Ma g^{-1} after the 80th cycle, the average charge capacity recovered to 615 mAh g^{-1}, slightly lower than the theoretical capacity for $NiMn_{1.7}Co_{1.8}O_4$. Galvanostatic cycling at high specific currents and the rate capability testing demonstrate that C-coated Ni-Mn-Co-O IOs have great potential to be a high rate anode material for Li-ion batteries. Figure S8b demonstrates that with increasing discharge and charge rates, coulombic efficiency remains remarkably stable in these binder and additive free IO anode materials once the final triple metal oxide system is formed.

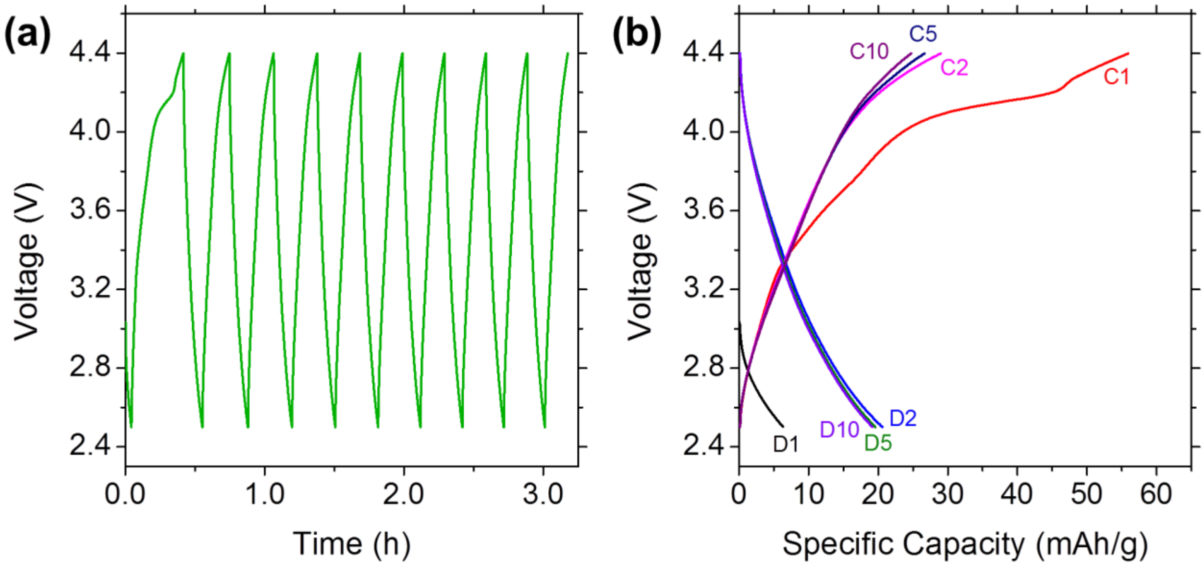

Figure S9. Electrochemical performance of Ni-Mn-Co-O IOs cycled in a cathode potential window (4.4-2.5 V). (a) Voltage time curves for the first 10 cycles, (b) Charge and discharge voltage profiles for the 1st, 2nd, 5th, and 10th cycles.

Table S2. Comparison of capacity values reported in this work with literature values

Material	Specific Current (mA g^{-1})	Charge Capacity mAh g^{-1}						Ref.
		1st	5th	10th	15th	20th	25th	
C-Coated Ni-Mn-Co-O IO	150	2414	1716	1341	1133	1000	914	This work
$MnCo_2O_4$ Microspheres	200	1008	914	868	822	764	706	20
$MnCo_2O_4$ hollow spheres	200	1471	959	920	860	784	742	41
$CoMn_2O_4$ hollow spheres	200	1425	856	810	781	735	709	41
Core–Shell Ellipsoidal $MnCo_2O_4$	100	892	874	892	907	948	904	60
$MnCo_2O_4$ microspheres	200	1131	352	292	263	260	260	65
$NiMn_2O_4$	100	633	450	377	335	312	306	66

References (for Supplementary Information)

1. Liu, Z.-Q. *et al.* Fabrication of Hierarchical Flower-like Super-Structures Consisting of Porous $NiCo_2O_4$ Nanosheets and their Electrochemical and Magnetic Properties. *RSC Adv*, **3**, 4372-4380, (2013).
2. Nguyen, T. *et al.* Structural Evolution, Magnetic Properties and Electrochemical Response of $MnCo_2O_4$ Nanosheet Films. *RSC Adv.* **5**, 27844-27852, (2015).
3. Steven, E. *et al.* Carbon Nanotubes on a Spider Silk Scaffold. *Nat. Commun.* **4**, 2435, (2013).
4. Díaz, C., Valenzuela, M. L., Lavayen, V. & O'Dwyer, C. Layered Graphitic Carbon Host Formation during Liquid-free Solid State Growth of Metal Pyrophosphates. *Inorg. Chem.* **51**, 6228-6236, (2012).
